# Modified subcostal approach to anterior quadratus lumborum block for managing postoperative pain in patients undergoing open nephrectomy

**DOI:** 10.1186/s44158-023-00102-w

**Published:** 2023-06-07

**Authors:** Cengiz Kaya, Burhan Dost, Hilal Dokmeci, Yasemin Burcu Ustun, Fatih Ozkan

**Affiliations:** Department of Anesthesiology and Reanimation, Ondokuz Mayis University Faculty of Medicine, Samsun, Turkey

**Keywords:** Quadratus lumborum block, Nephrectomy, Nerve blocks, Regional anesthesia, Postoperative pain, Ultrasonography, Diaphragm

## Abstract

**Background:**

Quadratus lumborum block is a relatively new truncal block and different approaches to this block have been described. With a recent modification to the subcostal approach to the anterior quadratus lumborum block (QLB3), the injection point was moved further cranially and medially, thereby aiming to enhance the spread of the local anesthetic into the thoracic paravertebral space. Although the level of blockade achieved with this modification seems sufficient for open nephrectomy, the modification is still for clinical evaluation. In this retrospective study, we aimed to evaluate the effects of the modified subcostal QLB3 approach on postoperative analgesia.

**Methods:**

All adult patients who received a modified subcostal QLB3 for postoperative analgesia following open nephrectomy between January 2021- 2022 were retrospectively evaluated. Accordingly, total opioid consumption and pain scores during rest/activity within the first 24 h after surgery were evaluated.

**Results:**

A total of 14 patients underwent open nephrectomy were analyzed. Pain scores within the first 6 h postoperatively, particularly the dynamic numeric rating scale (NRS) scores (4–6.5/10), were high. The median (interquartile range) resting and dynamic NRS scores for the first 24 h were 2.75 (1.79) and 3.91 (1.67), respectively. The mean ± standard deviation IV-morphine equivalent dose for the first 24 h was 30.9 ± 10.9 mg.

**Conclusions:**

It was found that the modified subcostal QLB3 did not provide satisfactory analgesia in the early postoperative period. Further randomized studies that extensively investigate the postoperative analgesic efficacy are required to draw a stronger conclusion.

## Introduction

Open nephrectomy causes a significant level of postoperative pain, and thoracic epidural analgesia is routinely used to treat this type of pain. However, the development of hypotension as a result of bilateral sympathectomy due to this technique limits its use. In addition, comorbidities and medications used in the patient population undergoing surgery, particularly anticoagulants, render the use of neuraxial techniques unfavorable [1].

Quadratus lumborum block (QLB) is among the relatively new truncal interfascial blocks that involve different local anesthetic (LA) injection sites and therefore different levels of dermatomal blockade [2]. Anterior QLB (QLB3) was first described by Borglum et al. in 2013, wherein the LA is injected between the quadratus lumborum and psoas major muscles at the L4 level [3]. Subcostal approach to the QLB3 was described by Elsharkawy in 2016. Here, the LA is instead injected between the investing fascia of the quadratus lumborum muscle (QLM) and anterior thoracolumbar fascia (ATLF) at 6–8 cm distance from the midline at L1/2 level [4]. However, it is not always possible to administer an injection between the investing fascia of the QLM and ATLF in practice. This stems from the challenge in distinguishing between these two structures by ultrasound, especially in obese patients. In addition, the situation becomes more complex, considering the fact that ATLF can be fused with the renal fascia and investing fascia [5].

Injecting the LA closer to the region where QLM attaches to the 12th vertebra makes it easier for the LA to reach the lower thoracic paravertebral space (TPVS), thereby ensuring a higher level of sensory block encompassing the mid-thoracic dermatomes. Indeed, in previous studies, dermatomal spread to T9–L1 was reported with QLB3 and to T6–L2 with the subcostal approach [3, 4]. However, cadaver studies failed to show a similar degree of spread, wherein the injectate could reach T10 level with QLB3 and T7/8 level with the subcostal approach [6, 7]. Li et al. described a modification based on these anatomical facts. Accordingly, acoustic shadow of the 12th rib, the diaphragm, lateral arcuate ligament (LAL), and QLM can be visualized by placing the ultrasound probe on the transverse process of L1 in the sagittal plane. Here, the LA is injected into the caudal aspect of LAL. Considering the mechanism of action of the block, the proximity of LA injection to LAL is directly proportional to the degree of LA spread into the TPVS. Hence, the authors reported that they could block T6–L1 dermatomes within just 10 min with 20 ml of 0.5% ropivacaine [5]. With this level of blockade, modified subcostal QLB3 can be used for analgesia in abdominal surgeries. To test this, we retrospectively evaluated the analgesic efficacy of the modified subcostal QLB3 in patients undergoing open nephrectomy.

## Method

A retrospective evaluation of the open nephrectomy case series was performed for the time period between January 2021 and January 2022 upon approval (2022/578) from the Clinical Trials Ethics Committee of Ondokuz Mayıs University. All adult patients who received modified subcostal QLB3 for postoperative analgesia were included in the study. The data were obtained from the medical anesthesia records of the university hospital. The data included patient descriptive characteristics and the numeric rating scale (NRS) scores during rest and activity, and heart rate and mean arterial pressure. The pain scores during rest and activity within the first 24 h postoperatively (post-anesthesia care unit [PACU]-3–6-12–18-24 h) according to NRS. Pain was managed with intravenous (IV) patient-controlled analgesia (PCA) (demand dose 20 µg/kg morphine, lockout period 6–10 min, 4-h limit equals 80% of the total calculated dose). Patients could demand opioids from the PCA device when the NRS score was ≥ 4 during activity. Conversely, patients who still had an NRS score ≥ 4 despite opioids were administered IV tramadol 100 mg for rescue analgesia. In addition, all patients were administered IV paracetamol 15 mg/kg in 6-h intervals. Total opioid consumption within the first 24 h starting from the PACU was recorded.

### Modified subcostal QLB3 technique

The patients were placed in sitting position and the relevant surgical field was prepared with povidone iodine according to the principles of asepsis/antisepsis followed by sterile draping. Convex ultrasound probe with a plastic sterile cover (2–5 MHz, GE LOGIQ V1 Ultrasound System, China) was placed 6–8 cm lateral to the L1 spinous process, with the orientation marker cranially located. Here, the acoustic shadow of the 12th rib and QLM were identified. The probe was then moved medially until the edge of the L1 transverse process was visible. The apposition zone between the QLM and the diaphragm and the acoustic shadow of the 12th rib were viewed on the ultrasound. The apposition zone enables spread of the LA to the TPVS through the inferior aspect of the lateral arcuate ligament and along the sub-endothoracic space. Visualization of the QLM, LAL, ATLF, and diaphragm movements was considered necessary for optimal ultrasound imaging.

**Fig. 1:**
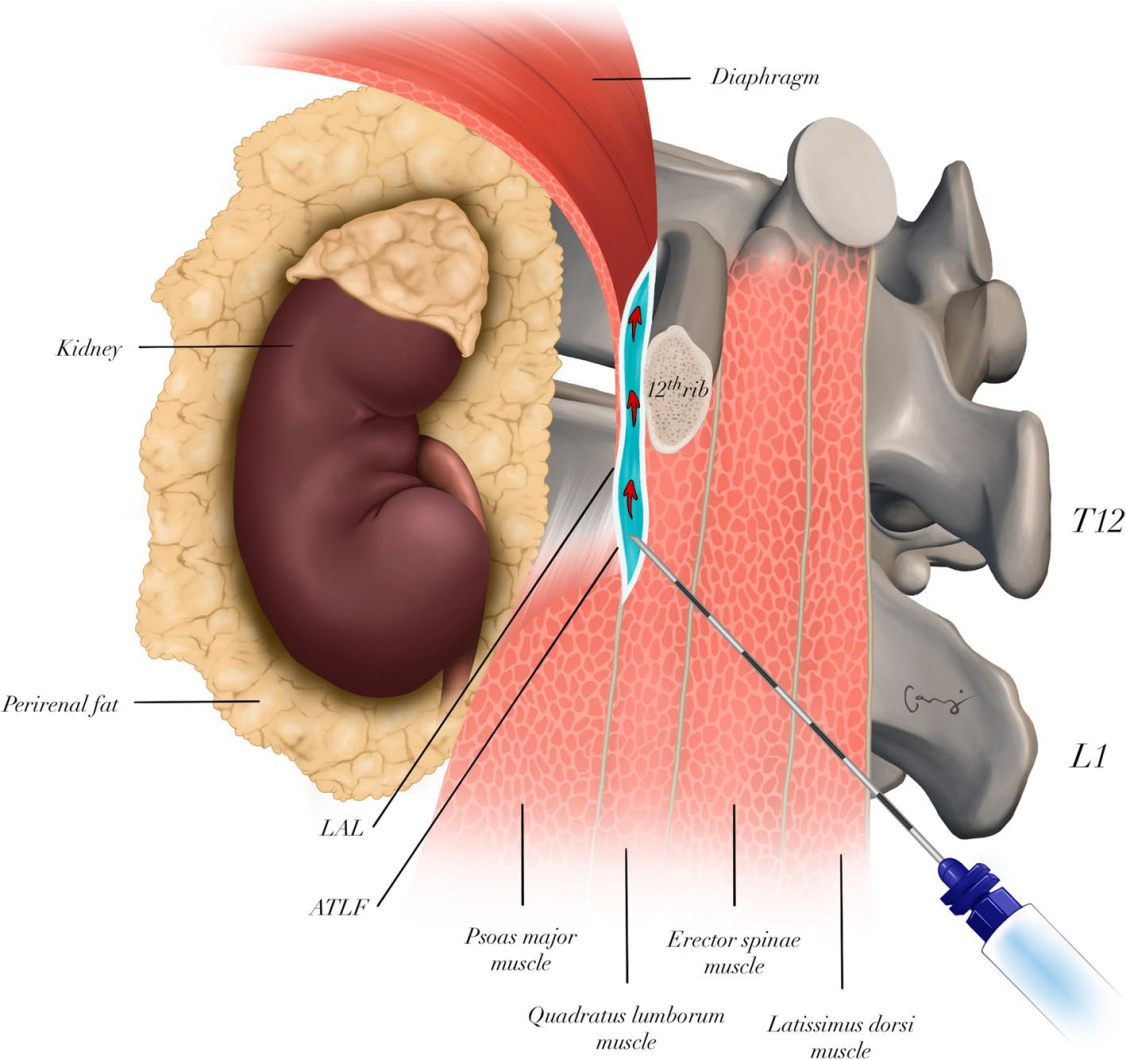
The sagittal schematic diagram shows the needle trajectory in the modified subcostal QLB3 and the cranial distribution of the LA. The LA passes posterior to the LAL and spreads to the lower thoracic paravertebral space. Abbreviations: QLB, quadratus lumborum block; LA, local anesthetic; LAL, lateral arcuate ligament; ATLF, anterior thoracolumbar fascia

**Fig. 2:**
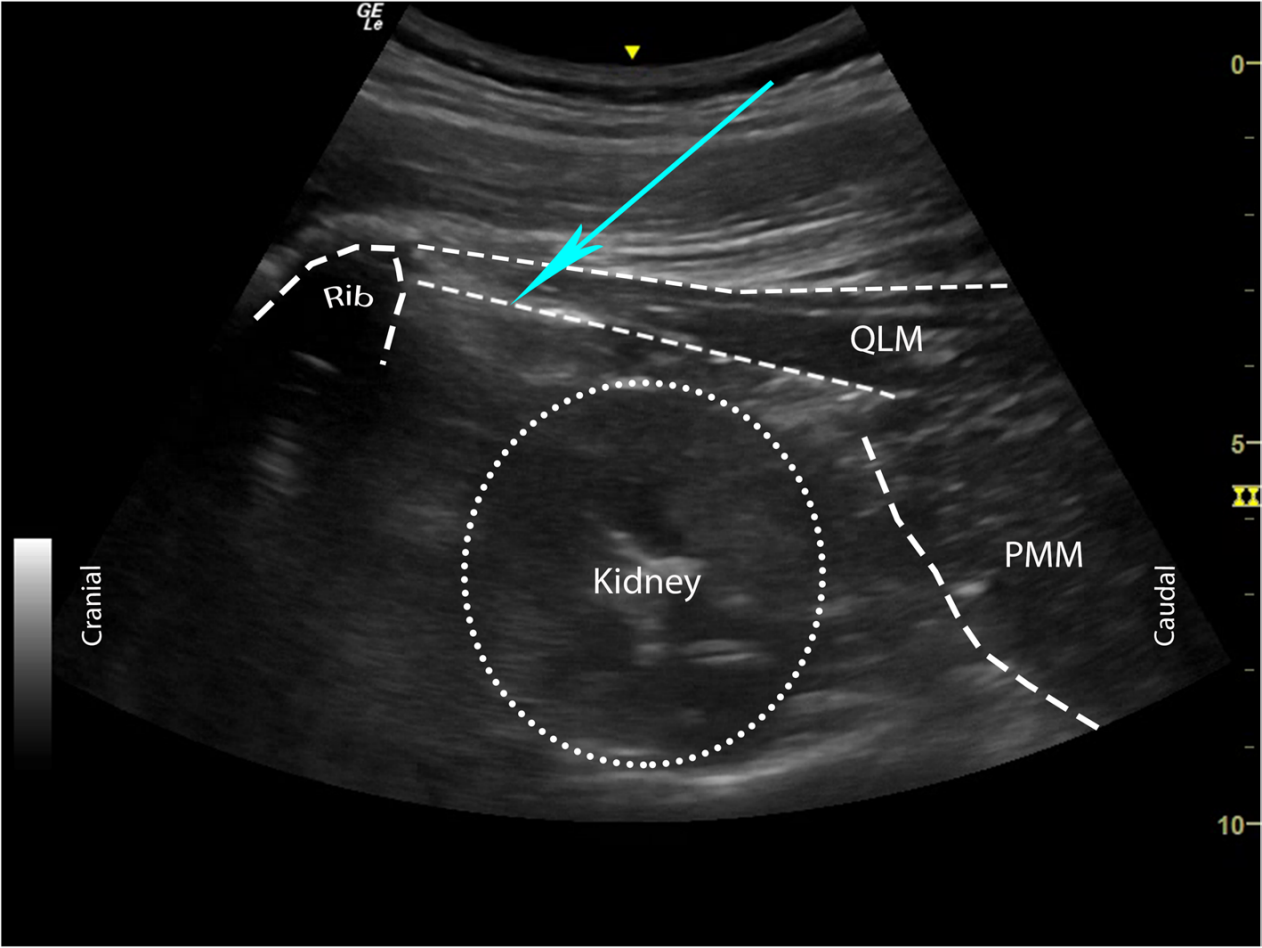
The sonographic landmarks of the modified subcostal QLB3 technique are depicted in an ultrasound image. The arrow indicates caudal-to-cranial needle trajectory. Abbreviations: QLB3, anterior quadratus lumborum block; QLM, quadratus lumborum muscle; PMM, psoas major muscle

For local infiltration, 1–2 ml of 1% lidocaine (Vem Pharmaceuticals, Turkey) was administered to the cutaneous-subcutaneous tissues where the block needle would be inserted. This was followed by moving a 21-G, 100-mm short-bevel block needle (Stimuplex® Ultra 360® by B. Braun, Germany) towards the plane between the investing fascia of QLM and ATLF right under the LAL in the caudocranial direction with the in-plane technique; 1–2 ml of 0.9% normal saline was injected to confirm the location of the needle (Figs. 1 and 2). After negative aspiration, 30 ml of 0.25% bupivacaine (Marcaine®, Astra Zeneca, Turkey) + 1:400.000 adrenaline solution was injected. Block success was defined as the observation of spread (lunar-shaped) of the LA through the inferior aspect of the LAL in the cranial direction throughout the sub-endothoracic space in real-time while administering the injection. Modified subcostal QLB3 was administered at the discretion of the anesthesiologist in the block room preoperatively.

### Anesthesia management

After patients were taken into the operating room, the anesthesia induction was performed with propofol (1–2 mg/kg) and remifentanil (infused at a rate of 0.1–0.25 mcg/kg/min), and endotracheal intubation was performed with rocuronium (1.2 mg/kg). Maintenance anesthesia was achieved with 1 MAC (minimum alveolar concentration) age-adjusted sevoflurane and O2/Air (fraction of inspired oxygen: 0.40) mixture, and remifentanil IV infusion (0.1–0.25 mcg/kg/min). After radial artery cannulation on the dependent side, the remifentanil infusion rate was adjusted to maintain heart rate and blood pressure within ± 20% of baseline values. At the end of the surgery, rocuronium was reversed with a combination of atropine and neostigmine, and extubation was performed. For analgesic purposes, IV tenoxicam 20 mg was administered after induction, and IV paracetamol 15 mg/kg was administered 20 min before the end of the case. For postoperative nausea and vomiting (PONV) prophylaxis, the patients were routinely administered IV dexamethasone 8 mg before induction and IV ondansetron 0.1 mg/kg 20 min before the end of the procedure. PONV was evaluated with a 4-point scale (0 = none, 1 = nausea without vomiting, 2 = one episode of vomiting, 3 = more than one episode of vomiting), and patients with a score > 1 were additionally administered IV ondansetron 0.1 mg/kg. If it failed, IV metoclopramide 0.15 mg/kg was added.

#### Statistical analysis

NRS scores were calculated for two distinct conditions, that is, during rest and activity, in the first 24 h postoperatively (PACU-3–6-12–18-24 h). In addition, total opioid consumption was recorded in the first 24 h starting from the PACU and converted to IV morphine equivalent dose (MED). The data were analyzed using the Statistical Package for the Social Sciences Version 24 (IBM, Armonk, NY, USA). The normality of the data was evaluated using the Shapiro–Wilk test. The quantitative data with a normal distribution were represented using mean ± standard deviation values, while the data with non-normal distribution were reported using median values (interquartile range). Categorical data were presented using absolute frequencies and percentages.

## Result

**Fig. 3:**
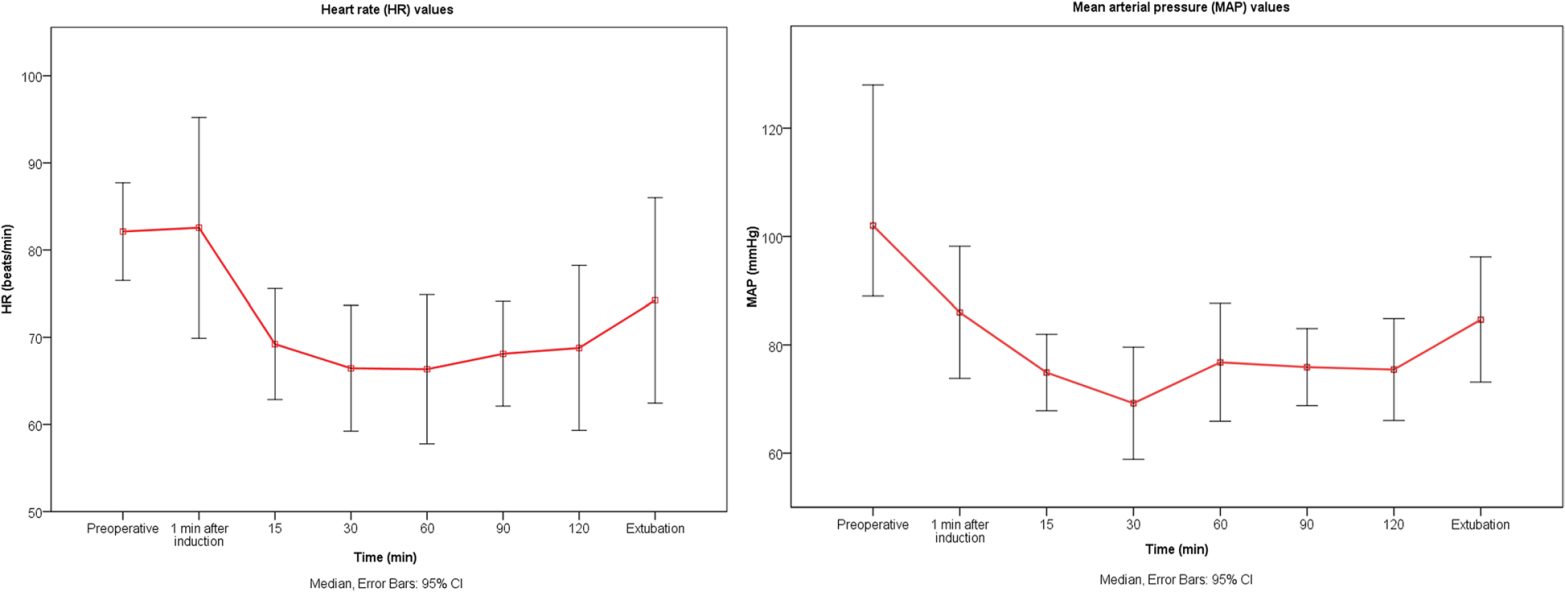
Intraoperative hemodynamic values

This case series consisted of 14 patients, that is, 7 patients (50%) who underwent open radical nephrectomy and 7 patients (50%) who underwent partial nephrectomy. Patient demographics and NRS scores during rest and activity were provided in Tables 1 and 2, and heart rate and mean arterial pressure values in Fig. 3. The median (IQR) resting/dynamic NRS scores for the first 24 h were 2.75 (1.79) and 3.91 (1.67), respectively. The mean ± standard deviation IV-MED for the first 24 h was 30.9 ± 10.9 mg. In terms of complications, no opioid or block-related complications were encountered in our patients, and only two patients received additional antiemetic treatment during the postoperative period.

**Table 1:** Demographic and surgical characteristics

	**Modified Subcostal QLB3** **(*n* = 14)**
**Age (years)**	61.1 ± 10.6
**Gender (Male/Female), n (%)**	12 (85.8) / 2 (14.2)
**ASA (II/III/IV), n (%)**	1 (7.1) / 12 (85.6) /1 (7.1)
**BMI (kg/m**^**2**^**)**	28 ± 2.9
**Surgery type**	
**Radical/ Partial n (%)**	7 (50) / 7 (50)
**Surgery time (min)**	132.9 ± 36.5
**Remifentanil (mcg)**	704.6 ± 262.3
**MED (mg)**	30.9 ± 10.9

Continuous variables are presented as mean ± standard deviation and categorical variables are presented as counts (percentages)

*Abbreviations*: *QLB* Quadratus Lumborum Block, *ASA* American Society of Anesthesiologists, *BMI* Body Mass Index, *MED* Morphine Equivalent Dose

**Table 2:** Postoperative static and dynamic NRS scores of patients at different times

	**Modified Subcostal Anterior QLB** **(*n* = 14)**	
	**Static NRS scores**	**Dynamic NRS scores**
**PACU**	5 (3)	6.5 (3)
**3th h**	4 (2)	5 (2)
**6th h**	3 (1)	4 (1)
**12th h**	2 (1)	3 (1)
**18th h**	1 (1)	3 (1)
**24th h**	1 (1)	2 (1)

Continuous variables are presented as median (interquartile range)

*Abbreviations*: *QLB* Quadratus Lumborum Block, *NRS* Numeric Rating Scale, *PACU* Post Anesthesia Care Unit

## Discussion

In patients undergoing open nephrectomy who received modified subcostal QLB3 with a single injection in the preoperative period, pain scores within the first 6 h post-surgery, especially the dynamic NRS scores, were high (4–6.5/10). However, the total 24-h pain scores were more reasonable.

Li et al. stipulated that the spread of LA to the TPVS would be increased by moving the injection point further cranially and medially with this modification of subcostal QLB3 [5]. In a descriptive study by Elsharkawy performed after describing subcostal QLB3, this block was administered with the continuous technique in patients undergoing open nephrectomy and renal transplantation [8]. The value of oral MED (mean ± SD) for the first 24 h was found to be 219.2 ± 183.5 mg, respectively. Despite the continuous technique used by the authors, the oral MED value (219.2 ± 183.5 vs. 92.7 ± 32.6 mg, *p* = 0.004) were lower in our patients compared to the values in the said study. Increased LA spread to the lower TPVS as a result of this modification may have led to this outcome. In the above-mentioned study, the patients had high pain scores in general and especially in the first day (4–5/10). The authors could mostly block the T8–12 dermatome (highest is T6; lowest is L2). This block level could have been insufficient for the pain after nephrectomy incision. Conversely, the authors attributed the mentioned high pain scores to the fact that three patients had chronic pain. In the present study, pain scores could not be reduced to the desired level in the early postoperative period. This may have stemmed from failure of the LA to reach the TPVS in a degree that would ensure splanchnic nerve/celiac ganglion blockade which is especially required for visceral analgesia [9].

In another randomized study conducted with patients undergoing open nephrectomy, continuous subcostal QLB3 was administered and the time-weighted average pain score for the first 72 h postoperatively and IV-MED value were found to be 4.7 ± 1.8 (mean ± SD) and 70 (43–125) mg (median [Q1-Q3]), respectively [10]. Similar to the descriptive study by Elsharkawy et al., this randomized study also reported higher pain scores and opioid consumption compared to our study. The fact that our patients exhibited lower values despite the use of the continuous technique in other studies can be considered an advantage of the modification.

In the literature, there are also case presentations that contradict our article. For instance, it was reported that a satisfactory level of analgesia was achieved with a single injection in patients undergoing nephrectomy who received QLB3, wherein one patient had 13 mg of 24-h morphine consumption and another patient did not even need additional analgesics [11, 12]. In addition, 48-h morphine consumption was found to be 24–79 mg, when continuous technique was used in 4 patients undergoing nephrectomy [13]. The authors argued that this effect was provided by the spread of the LA to the lower TPVS with an injection administered at the L4 level. However, a similar effect was not observed in the present study, even with an injection at the L1 level.

Although studies on QLB in laparoscopic nephrectomy are ongoing, the existing data are promising for postoperative pain control and opioid reduction in patients [14]. Compared with continuous epidural analgesia, Aditianingsih et al. found bilateral anterior QLB, and Rahendra et al. found lateral QLB provided equivalent analgesia in patients [15, 16]. Therefore, additional studies are needed to compare the modified subcostal approach with single injection/catheter technique to neuraxial techniques.

### Limitations

Firstly, the statistical value of our deductions is limited, since a small patient group was evaluated in this retrospective analysis. In addition, the present study was not a randomized but a descriptive study. Finally, the study did not include any obese patients.

This modification of the subcostal QLB3 should be compared with placebo or other classic QLB approaches in prospective randomized studies to be conducted in the future. This would enable a more extensive investigation into the postoperative analgesic efficacy of this technique. Moreover, it should be investigated in cadaver and imaging studies whether this modification increases LA spread into the lower TPVS.

## Conclusion

In summary, it was found that modified subcostal QLB3 below the lateral arcuate ligament level did not provide satisfactory analgesia in the early postoperative period.

## Data Availability

The datasets used and/or analysed during the current study available from the corresponding author on reasonable request.

## Abbreviations

QLB: Quadratus Lumborum Block
NRS: Numeric Rating Scale
IQR: Interquartile Range
QLM: Quadratus Lumborum Muscle
ATLF: Anterior Thoracolumbar Fascia
LA: Local Anesthetic
TPVS: Thoracic Paravertebral Space
LAL: Lateral Arcuate Ligament
PACU: Post-Anesthesia Care Unit
IV: Intravenous
PCA: Patient-Controlled Analgesia
MED: Morphine Equivalent Dose

## References

[1] Nimmo SM, Harrington LS (2014). What is the role of epidural analgesia in abdominal surgery?. Contin Educ Anaesth Crit Care Pain.

[2] Elsharkawy H, El-Boghdadly K, Barrington M (2019). Quadratus lumborum block: anatomical concepts, mechanisms, and techniques. Anesthesiology.

[3] Børglum J, Moriggl B, Jensen K, Lønnqvist PA, Christensen AF, Sauter A, et al (2013) Ultrasound-Guided Transmuscular Quadratus Lumborum Blockade. BJA Br J Anaesth 111 eLetters.

[4] Elsharkawy H (2016). Quadratus lumborum block with paramedian sagittal oblique (subcostal) approach. Anaesthesia.

[5] Li H, Shi R, Wang Y (2021). A modified approach below the lateral arcuate ligament to facilitate the subcostal anterior quadratus lumborum block. J Pain Res.

[6] Dam M, Moriggl B, Hansen CK, Hoermann R, Bendtsen TF, Børglum J (2017). The pathway of injectate spread with the transmuscular quadratus lumborum block: a cadaver study. Anesth Analg.

[7] Elsharkawy H, El-Boghdadly K, Kolli S, Esa WAS, DeGrande S, Soliman LM (2017). Injectate spread following anterior sub-costal and posterior approaches to the quadratus lumborum block. Eur J Anaesthesiol.

[8] Elsharkawy H, Ahuja S, DeGrande S, Maheshwari K, Chan V (2019). Subcostal approach to anterior quadratus lumborum block for pain control following open urological procedures. J Anesth.

[9] Akerman M, Pejčić N, Veličković I (2018). A review of the quadratus lumborum block and ERAS. Front Med.

[10] Elsharkawy H, Ahuja S, Sessler DI, Maheshwari K, Mao G, Sakr Esa WA (2021). Subcostal anterior quadratus lumborum block versus epidural block for analgesia in open nephrectomy: a randomized clinical trial. Anesth Analg.

[11] Gürkan Y (2017). Quadratus Lumborum Block for both cholecystectomy and right sided nephrectomy. Ağrı - J Turkish Soc Algol.

[12] Corso RM, Piraccini E, Sorbello M, Bellantonio D, Tedesco M (2017). Ultrasound-guided transmuscular quadratus lumborum block for perioperative analgesia in open nephrectomy. Minerva Anestesiol.

[13] Kadam V, Howell S (2018). Ultrasound-guided continuous transmuscular quadratus lumborum block- L4 or L2 level catheter insertion for analgesia in open abdominal surgery: Case series. Indian J Anaesth.

[14] Little C, Rahman S (2021). Quadratus lumborum blocks in nephrectomy: a narrative review. Local Reg Anesth.

[15] Aditianingsih D, Pryambodho, Anasy N, Tantri AR, Mochtar CA (2019). A randomized controlled trial on analgesic effect of repeated Quadratus Lumborum block versus continuous epidural analgesia following laparoscopic nephrectomy. BMC Anesthesiol.

[16] Rahendra R, Pryambodho P, Aditianingsih D, Sukmono RB, Tantri A, Melati AC (2019). Comparison of IL-6 and CRP concentration between quadratus lumborum and epidural blockade among living kidney donors: a randomized controlled trial. Anesth Pain Med.

